# Postlactational involution biomarkers plasminogen and phospho-STAT3 are linked with active age-related lobular involution

**DOI:** 10.1007/s10549-017-4413-3

**Published:** 2017-07-27

**Authors:** Melody L. Stallings-Mann, Ethan P. Heinzen, Robert A. Vierkant, Stacey J. Winham, Tanya L. Hoskin, Lori A. Denison, Aziza Nassar, Lynn C. Hartmann, Daniel W. Visscher, Marlene H. Frost, Mark E. Sherman, Amy C. Degnim, Derek C. Radisky

**Affiliations:** 1 Department of Cancer Biology, Mayo Clinic, Jacksonville, FL 32224 USA; 2 Department of Health Sciences Research, Mayo Clinic, Rochester, MN 55905 USA; 3 Department of Information Technology, Mayo Clinic, Rochester, MN 55905 USA; 4 Department of Laboratory Medicine and Pathology, Mayo Clinic, Jacksonville, FL 32224 USA; 5 Department of Medical Oncology, Mayo Clinic, Rochester, MN 55905 USA; 6 Department of Laboratory Medicine and Pathology, Mayo Clinic, Rochester, MN 55905 USA; 7 Department of Health Sciences Research, Mayo Clinic, Jacksonville, FL 32224 USA; 8 Department of Surgery, Mayo Clinic, Rochester, MN 55905 USA

**Keywords:** Lobular involution, Postlactational involution, Biomarkers, Breast cancer, Cohort studies

## Abstract

**Purpose:**

Breast terminal duct lobular units undergo two distinctive physiological processes of involution: age-related lobular involution (LI), which is gradual and associated with decreased breast cancer risk, and postlactational involution, which is relatively precipitous, occurs with weaning, and has been associated with potentiation of tumor aggressiveness in animal models. Here we assessed whether markers of postlactational involution are associated with ongoing LI in a retrospective tissue cohort.

**Methods:**

We selected 57 women from the Mayo Clinic Benign Breast Disease Cohort who underwent multiple biopsies and who were average age 48 at initial biopsy. Women were classified as having progressive or non-progressive LI between initial and subsequent biopsy. Serial tissue sections were immunostained for plasminogen, matrix metalloproteinase 9 (MMP-9), phospho-STAT3 (pSTAT3), tenascin C, Ki67, CD44, cytokeratin 14 (CK14), cytokeratin 19 (CK19), and c-myc. All but Ki67 were digitally quantified. Associations between maximal marker expression per sample and progressive versus non-progressive LI were assessed using logistic regression and adjusted for potential confounders.

**Results:**

While no biomarker showed statistically significant association with LI progression when evaluated individually, lower expression of pSTAT3 (OR 0.35, 95% CI 0.13–0.82, *p* = 0.01) and higher expression of plasminogen (OR 2.89, 95% CI 1.14–8.81, *p* = 0.02) were associated with progressive LI in models simultaneously adjusted for all biomarkers. Sensitivity analyses indicated that the strengthening in association for pSTAT3 and plasminogen with progressive LI was due to collinearity between these two markers.

**Conclusions:**

This is the first study to identify biomarkers of active LI. Our findings that plasminogen and pSTAT3 are significantly associated with LI suggest that they may represent signaling nodes or biomarkers of pathways common to the processes of postlactational involution and LI.

**Electronic supplementary material:**

The online version of this article (doi:10.1007/s10549-017-4413-3) contains supplementary material, which is available to authorized users.

## Introduction

Terminal duct lobular units (TDLUs) are microscopic structures comprising terminal ducts and acinar substructures that produce milk after birth and may develop cancer precursors among some women. With aging, TDLUs undergo age-related lobular involution (LI), such that the number and size of acini per TDLU are reduced and the intralobular stroma is replaced with collagen [1]. Analysis of the Mayo Clinic Benign Breast Disease (BBD) cohort of more than 13,000 women who underwent a surgical biopsy diagnosed as BBD showed that the timing of LI centers around the perimenopausal years, although there was considerable variation among women [2], and that incomplete LI among postmenopausal women or delays in the ongoing process of LI are associated with increased breast cancer (BC) risk [2, 3]. Such observations indicate that investigations to understand the biological processes underlying LI may reveal markers and mechanisms associated with lowering of breast cancer risk. However, very little is known about the signaling processes that control LI or why ongoing LI is associated with decreased BC risk.

Postlactational involution, which is triggered by weaning, is a distinct process from LI, involving a highly controlled collapse of alveolar structures, programmed removal of secretory epithelial cells, phagocytosis by macrophages, proteolytic degradation of basement membranes, and stromal remodeling [4]. As a result, most of the differentiated epithelial cells disappear and an adipocyte-rich stroma, in which the resting ductal system is embedded, reappears [5–9]. During postlactational remodeling, the mammary gland shares striking similarities with pathologically induced wound-healing and tumorigenic microenvironments, and the postlactational state is associated with increased cancer progression [10–12]. Moreover, experimental studies using animal models have shown that delays in postlactational lobular involution are associated with increased cancer incidence and progression [13].

Thus, since increased cancer incidence and progression have been associated with delays in age-related LI as well as delays in postlactational LI, we set out to determine whether mediators and effectors of postlactational remodeling are associated with the physiological process of age-related LI. We used tissue biopsies from the Mayo BBD multiple biopsy cohort, a group of women who had multiple biopsies with benign findings and for which LI status has been determined at initial and subsequent biopsy [3]. We compared two groups composed of women who showed LI progression between the initial to the subsequent biopsy, and those who did not. We stained sequential sections, using tissue from the initial benign biopsy, for a range of biomarkers that have been implicated in postlactational involution, including pSTAT3 and c-Myc, key modulators of apoptosis in the early stages of postlactational involution [7, 14, 15], the proliferation marker Ki67, which shows increased expression when postlactational involution is delayed [16]. We also evaluated expression of the matrix metalloprotease MMP9, which is activated during postlactational involution to induce remodeling of the extracellular matrix (ECM) [17–19], and the preprotease plasminogen, which is required for postlactational involution [20, 21]. We measured expression of ECM component Tenascin C and ECM receptor CD44, expressed during postlactational involution [22, 23] and markers of luminal (CK19) and myoepithelial (CK14) cells, which can show altered abundance during and after postlactational involution [24]. We assessed staining of all markers in individual TDLUs using unbiased digital quantification methods, and analyzed relative expression of each marker and its association with LI progression. Our analyses of these results revealed for the first time specific molecules that correlated with age-related LI status, and implicated functional pathways that may underlie both postlactational and LI involution processes.

## Methods

### Study population

The Mayo BBD Cohort has been described previously [2, 25] and currently comprises 13,455 women who had a benign breast biopsy at Mayo Clinic (Rochester, MN) from 1967 to 2001. Demographic information, clinical data, and breast cancer risk factors were identified from medical records and questionnaires [2, 25]. Within this cohort, 1115 women were identified who had undergone at least one additional benign biopsy more than 60 days after the initial biopsy (multiple biopsy cohort) and which occurred prior to any diagnosis of breast cancer [3]. To enrich for women expected to be perimenopausal on the basis of age at initial biopsy, we selected women from the multiple biopsy sub-cohort who were aged 40–58 at initial biopsy (exact menopause incidence is not available for most women in the BBD cohort), and who had a subsequent biopsy with benign findings. The study pathologist (DWV) microscopically reviewed hematoxylin and eosin-stained sections of benign biopsies to classify severity of benign breast disease (non-proliferative, proliferative disease without atypia, or atypical hyperplasia) [25, 26]. Level of LI was classified using a four-level qualitative scale (0–25% involuted, 26–50%, 51–75%, and >75%), to provide increased resolution in the LI process, and as described previously [3]. Change in LI status from the initial biopsy to the subsequent biopsy was defined as progressed (greater extent of involution at second biopsy) or non-progressive (equal or lesser extent of involution at second biopsy). Initially, 69 patient samples were selected for this study. Ten individuals with initial involution >75% TDLU at initial biopsy were eliminated since by definition they could not progress beyond that category. One individual was eliminated because her biopsy did not show interpretable immunohistochemical staining. Our final sample size was 57 individuals: 26 who progressed and 31 who did not progress. The study protocol was approved by the Mayo Clinic Institutional Review Board.

### Immunohistochemistry and digital analysis

For each study sample, serial formalin-fixed paraffin-embedded (FFPE) tissue sections from the initial biopsy were stained immunohistochemically using previously described methods [27]. The following immunostains were performed: CK19 (Neomarkers MS-1671P at 1:500), CK14 (Abcam ab7800 at 1:200), CD44 (DAKO 1485, which recognizes standard and all variable CD44 splice isoforms [28], at 1:25), MMP9 (Abcam ab38898 at 1:10,000), Tenascin C (Abcam ab6393 at 1:200), pSTAT3 (Abcam ab330646 at 1:200), c-Myc (Abcam ab32 at 1:500), Plasminogen (Santa Cruz Biotechnology sc-25546 at 1:200), and Ki67 (DAKO M72400 at 1:100). Negative controls are provided in Supplementary Fig. 1. Color deconvolution method for image analysis is described in Supplemental Methods.

### Statistical analysis

Data were summarized using means and standard deviations (SDs) for continuous variables, and frequencies and percentages for categorical variables, including histologic impression, year of initial biopsy, age at initial biopsy, time between initial and subsequent biopsy, family history of breast cancer, number of children/age at first birth, involution at initial biopsy, body mass index, presence of sclerosing adenosis, and ever use of hormone replacement therapy. We compared associations of demographic and clinical characteristics at initial biopsy with progression in involution status from index to second biopsy using t-tests for continuous variables and Chi-square tests for categorical variables.

Continuously distributed biomarker expression values were transformed using a probit (inverse normal) transformation to correct for inherent right skewness of the data and to ensure a common expression distribution scale across all biomarkers. This type of transformation yields values that can be interpreted roughly as t-statistics, that is, the number of standard deviations a given value deviates from the mean of the distribution of expression values on a standard normal curve. Ki67 expression, which was measured using a four-level ordinal scale instead of a continuous scale, was not transformed.

Associations between biomarkers and progression of involution were examined using logistic regression analysis, modeling progression as the outcome variable and biomarker expression as log-linear one degree-of-freedom predictor variables. Three sets of models were fit. First, we ran separate analyses for each biomarker, adjusting for year of biopsy, age at initial biopsy, and time between initial and second biopsy, as potential confounding variables. Next, we fit one overall model that simultaneously included all biomarkers and the same set of potential confounding variables. Third, we ran an AIC-based backward elimination stepwise model starting with all biomarkers and forcing in the potential confounding variables. This process removed at each step the variable which resulted in a model with lowest AIC, stopping when no such variable removal lowered the AIC.

A unique biomarker-specific expression value was recorded for each TDLU, resulting in multiple expression values per individual. Primary analyses modeled the maximum of all TDLU expression values per individual as the predictor variable, since we hypothesized that the LI process could be occurring at different rates in different TDLU within a single individual, and that the TDLU with maximal biomarker expression would best reflect this process. We also performed secondary analyses that used the median of all such values. The functional forms of the associations between biomarker expression and progression were assessed using natural cubic splines with corresponding 95% confidence bands [29]. All statistical tests were two-sided, and all analyses were carried out using R version 3.3.1.

## Results

### Characteristics of subjects and tissue samples

**Fig. 1:**
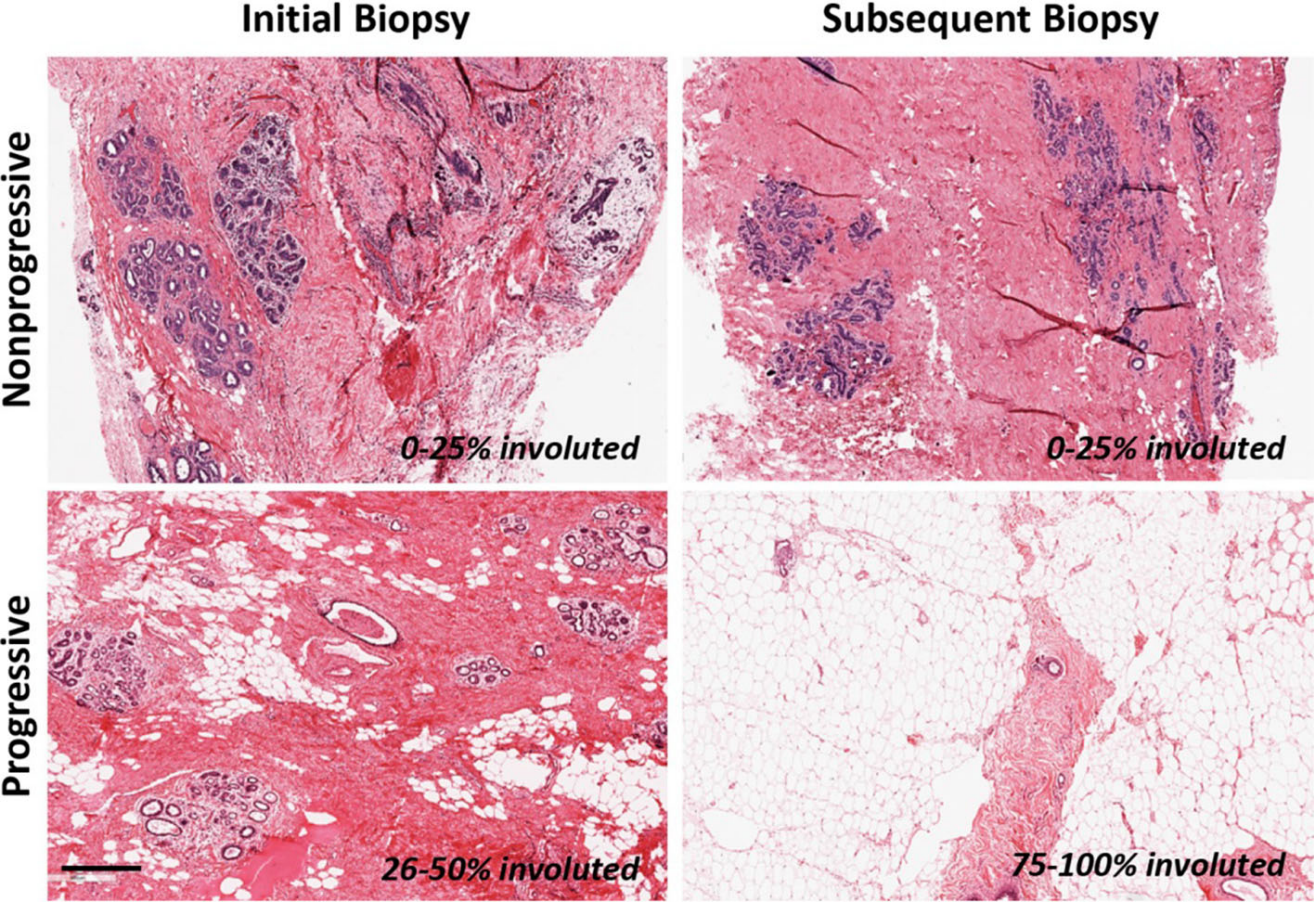
Representative images of benign breast biopsies from both the initial biopsy and the subsequent biopsy. An example of non-progressive LI and progressed LI are shown. All images are at the same magnification. *Scale bar* 400 µm

Of the 13,455 patients in the Mayo BBD cohort, 1115 were previously found to have had multiple biopsies, as previously reported [3]. As the timing of LI centers around the perimenopausal years [1, 2, 30], we enriched for women expected to be in the active process of age-related LI by selecting a set from the multiple biopsy group that were 40–58 years of age at initial biopsy, with an average age of 48.3 years, resulting in a final sample size of 57 women: 31 non-progressors (no change in LI status between biopsies, 54%) and 26 progressors (increase in LI from initial biopsy to subsequent biopsy, 46%) (Fig. 1). Comparisons of demographic and clinical variables with progression of involution are provided in Table 1. No other variables were significantly or marginally associated with progression (*p* > 0.10 for all).

**Table 1:** Associations of LI progression with demographics and clinical variables (*N* = 57)

	Did not progress (*N* = 31)	Progressed (*N* = 26)^a^	*p* value^b^
Age at initial biopsy			0.573
Mean (SD)	48 (5.06)	48.8 (5.62)	
Time between biopsies (years)			0.844
Mean (SD)	2.59 (1.22)	2.52 (1.37)	
LI at initial biopsy			0.230
0–25% TDLU	6 (19.4%)	10 (38.5%)	
26–50% TDLU	10 (32.3%)	8 (30.8%)	
51–75% TDLU	15 (48.4%)	8 (30.8%)	
LI at subsequent biopsy			<0.001
0–25% TDLU	7 (22.6%)	0 (0%)	
26–50% TDLU	14 (45.2%)	5 (19.2%)	
51–75% TDLU	10 (32.3%)	10 (38.5%)	
>75% TDLU	0 (0%)	11 (42.3%)	
Histologic impression			0.427
AH	4 (12.9%)	4 (14%)	
NP	11 (35.5%)	13 (42.1%)	
PDWA	16 (51.6%)	9 (43.9%)	
Family history of breast cancer			0.483
None	20 (64.5%)	13 (50%)	
Weak	7 (22.6%)	7 (26.9%)	
Strong	4 (12.9%)	6 (23.1%)	
Age at 1st live birth/#children			0.386
<21, 1 or more	6 (26.1%)	5 (21.7%)	
≥21, 1–2	3 (13%)	8 (34.8%)	
≥21, 3 or more	11 (47.8%)	8 (34.8%)	
Nulliparous	3 (13%)	2 (8.7%)	
Body mass index at initial biopsy			0.893
≤21	3 (13.6%)	2 (9.52%)	
22–25	10 (45.5%)	12 (57.1%)	
26–29	5 (22.7%)	4 (19%)	
30+	4 (18.2%)	3 (14.3%)	
Sclerosing adenosis			1.000
Absent	14 (45.2%)	12 (46.2%)	
Present	17 (54.8%)	14 (53.8%)	
Use of HRT			0.158
Never	4 (17.4%)	9 (40.9%)	
Ever	19 (82.6%)	13 (59.1%)	

*NP* non-proliferative disease, *PDWA* proliferative disease without atypia, *AH* atypical hyperplasia, *SD* standard deviation, *TDLU* terminal duct lobular units

^a^Values represented as *N* (percent) unless otherwise indicated

^b^Chi-square test for categorical variables, *t* test for continuous variables

### Protein expression of postlactational involution markers in breast tissue

**Fig. 2:**
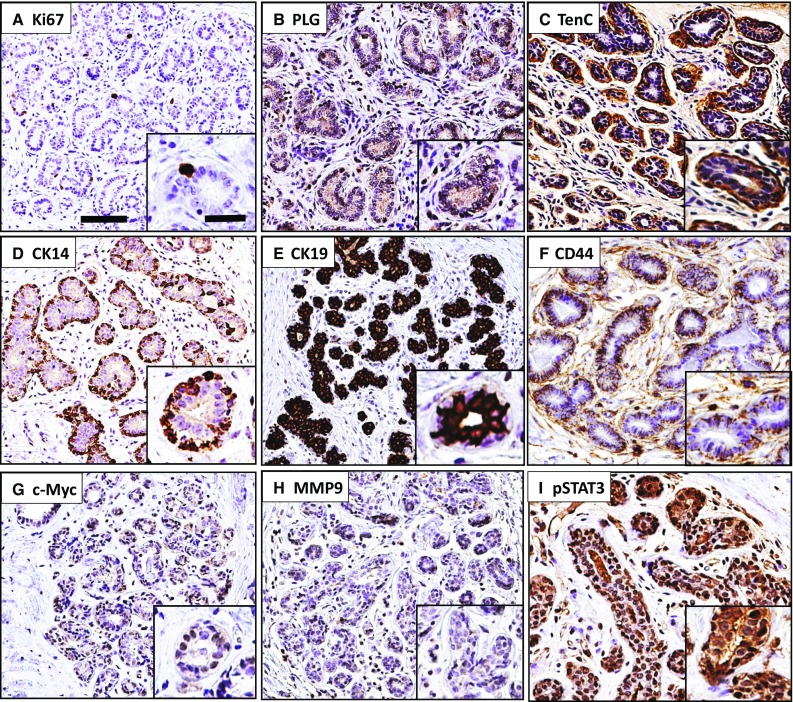
Representative images of each marker. All images are at the same magnification. *Scale bar* large insets, 100 µm; insets, 30 mm

Based upon qualitative review of histologic images, we observed characteristic staining patterns of each postlactational involution marker in breast tissue. Nuclear expression of the proliferation marker Ki67 was observed primarily in epithelial cells (Fig. 2a). Immunopositivity for plasminogen, precursor of the proteolytic molecule plasmin, was found primarily at the membrane in epithelial cells (Fig. 2b). Expression of the extracellular matrix glycoprotein tenascin C (TNC) was abundant in the interlobular and/or intralobular stroma (Fig. 2c). As expected, cytokeratin 14 (CK14) was expressed strongly in myoepithelial cells (Fig. 2d) and cytokeratin 19 (CK19) was expressed in luminal epithelial cells (Fig. 2e). CD44, a cell-surface glycoprotein involved in cell–cell interactions, cell adhesion and migration, was found to stain both myoepithelial and luminal epithelial cells (Fig. 2f). Immunostaining of the c-myc transcription factor was present within the nuclei or the cytoplasm of epithelial cells and stromal cells (Fig. 2g). Most TDLUs were negative for the secreted matrix metalloproteinase MMP-9, but when present, faint signal was detected in the cytoplasm of luminal cells, myoepithelial cells, and stromal cells (Fig. 2h). The signal transducer and transcriptional activator STAT3 is phosphorylated in response to growth factors and shuttles between the cytoplasm and nucleus, and phospho-STAT3 (pSTAT3) was found abundantly expressed in the epithelial and stromal components of breast tissue (Fig. 2i).

**Fig. 3:**
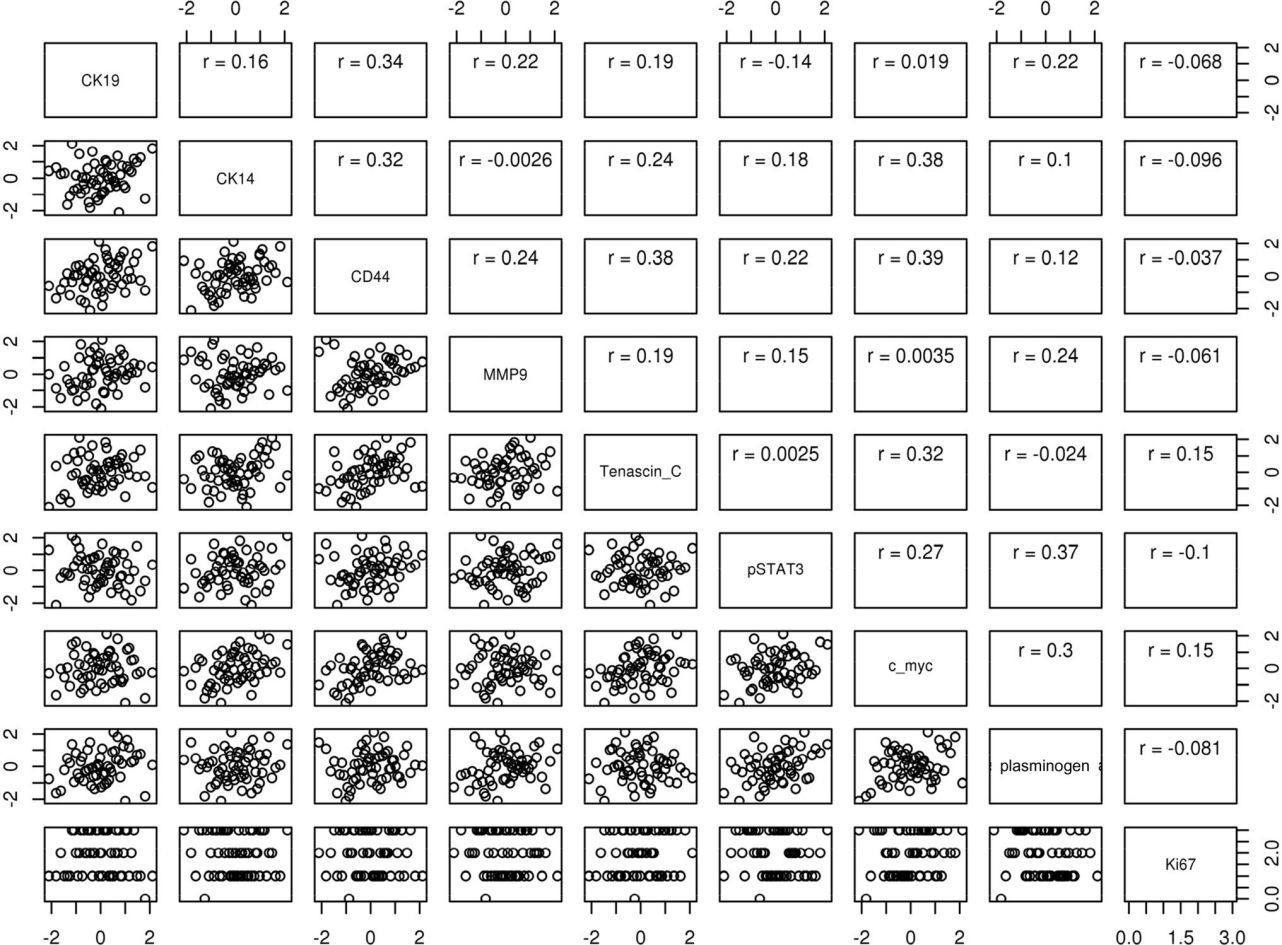
Pairwise correlations of biomarker expression values, based on maximum expression across all TDLU measured within a person. All biomarker values except for Ki67 were transformed using a probit (inverse normal) transformation

Pairwise correlations of biomarker expression, using the maximum of all biomarker-specific values per individual, are presented in Fig. 3. We observed mild to moderate correlations for most pairs of biomarkers.

### Progression of involution is associated with lower expression of pSTAT3 and higher expression of plasminogen

**Fig. 4:**
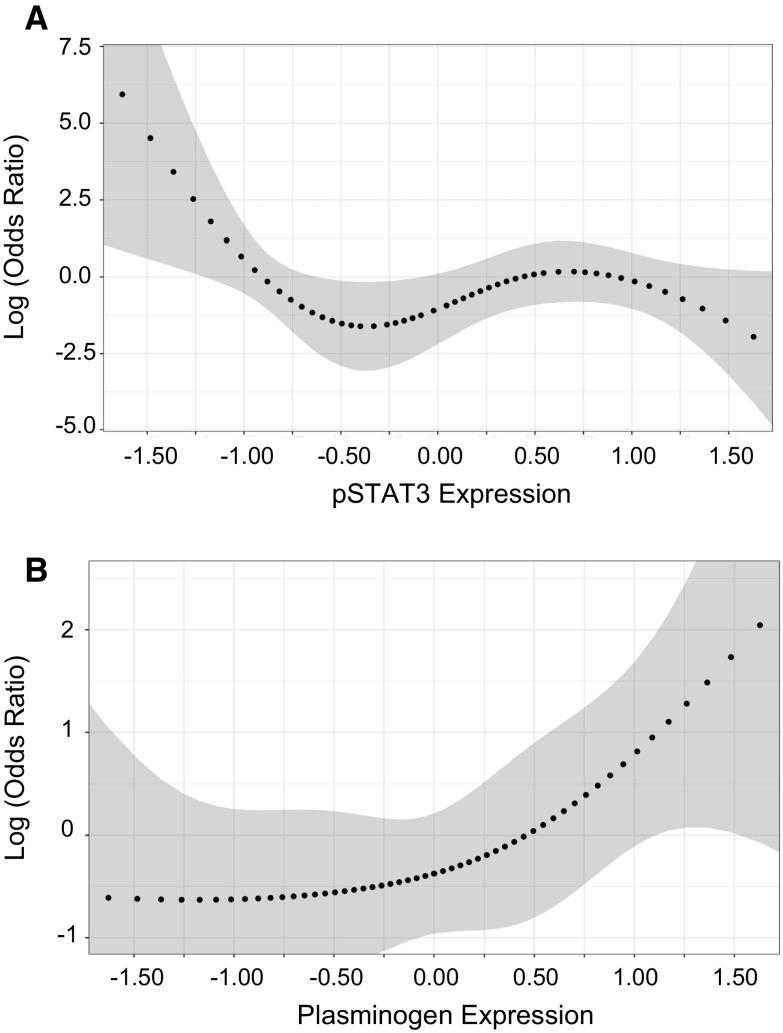
Natural cubic splines for association of progression of involution with biomarker expression. *Gray bands* indicate 95% confidence. Analyses are adjusted for year of initial biopsy, age at initial biopsy, and time between initial and subsequent biopsies. Panel A displays pSTAT3 and Panel B displays plasminogen

Comparisons of progression of involution with biomarker expression, using the maximum of all biomarker-specific values per individual, are provided in Table 2. After adjustment for year of initial biopsy, age at initial biopsy and time between biopsies, we found that progression was associated with lower expression of pSTAT3, such that the odds of progression decreased by 48% for each 1 standard deviation increase in the probit-transformed pSTAT3 expression (OR 0.52, 95% CI 0.25–0.99, *p* = 0.05). Conversely, progression was positively associated with higher expression of Plasminogen, in that the odds of progression doubled for each standard deviation increase in the transformed expression value (OR 2.06, 95% CI 1.01–4.61, *p* = 0.05). Images depicting varying expression of pSTAT3 and Plasminogen expression are provided in Supplementary Fig. 2; staining differences in representative patients displaying progressive vs non-progressive LI are provided in Supplementary Fig. 3. No other biomarkers demonstrated an association with progression. In the adjusted logistic regression model that simultaneously included all expression biomarkers, the associations of progression with pSTAT3 (OR 0.35, 95% CI 0.13–0.82, *p* = 0.02) and plasminogen (OR 2.89, 95% CI 1.14–8.81, *p* = 0.03) strengthened further. Sensitivity analyses indicated that this strengthening in association for pSTAT3 and plasminogen was specifically due to associations between these two variables: adjusted logistic regression models that simultaneously included all expression biomarkers except plasminogen did not show strengthened association for pSTAT3 (OR 0.48, 95% CI 0.20–1.05, *p* = 0.07), and similar analyses that excluded pSTAT3 did not show strengthened association for plasminogen (OR 1.87, 95% CI 0.85–4.41, *p* = 0.12). Multivariate analyses that included LI status at initial biopsy were performed as an additional sensitivity analysis, but the results were similar: plasminogen is still associated with increased likelihood of LI progression (OR 2.83, 95% CI 1.03–9.47) and pSTAT3 is still associated with decreased likelihood of LI progression (OR 0.41, 95% CI 0.14–1.03). Cubic splines visually depicting associations of pSTAT3 and plasminogen are presented in Fig. 4. pSTAT3 exhibited declines in the odds of progression at the tails of the expression distribution, with a leveling of the odds in the middle of the distribution, whereas plasminogen demonstrated a monotonically positive association with progression.

**Table 2:** Associations of progression of involution with biomarker expression, based on maximum expression across all TDLU measured within an individual

Biomarker	Did not progress (*N* = 31) mean (SD)^a^	Progressed (*N* = 26) mean (SD)^a^	OR (95% CI)^a,b^	*p* value^a,b^	OR (95% CI)^a,c^	*p* value^a,c^
CK19	0.027 (1.07)	−0.032 (0.807)	1.33 (0.71–2.59)	0.38	0.96 (0.42–2.16)	0.91
CK14	−0.102 (0.864)	0.121 (1.04)	0.83 (0.43–1.58)	0.57	0.97 (0.42–2.19)	0.94
CD44	−0.18 (0.928)	0.215 (0.943)	0.80 (0.39–1.58)	0.52	1.13 (0.47–2.79)	0.78
MMP9	−0.129 (0.841)	0.154 (1.06)	0.90 (0.46–1.75)	0.75	0.90 (0.40–2.02)	0.80
Tenascin C	−0.193 (0.905)	0.223 (0.962)	0.73 (0.36–1.42)	0.35	0.86 (0.37–1.98)	0.72
pSTAT3	−0.215 (1)	0.256 (0.823)	0.52 (0.25–0.99)	0.05	0.35 (0.13–0.82)	0.01
c-Myc	−0.142 (1.05)	0.169 (0.795)	0.86 (0.44–1.69)	0.67	1.00 (0.39–2.52)	0.99
Plasminogen	0.123 (1.06)	−0.146 (0.783)	2.06 (1.01–4.61)	0.05	2.89 (1.14–8.81)	0.02
Ki67	1.9 (0.944)	2 (0.849)	0.70 (0.34–1.36)	0.30	0.60 (0.24–1.35)	0.22

*SD* standard deviation, *OR* odds ratio, *CI* confidence interval

^a^All biomarker values except Ki67 were transformed using a probit (inverse normal) transformation

^b^Adjusted for year of initial biopsy, age at initial biopsy, and time between initial and subsequent biopsy

^c^Adjusted for year of initial biopsy, age at initial biopsy, time between initial and subsequent biopsy, and all other biomarkers in the table

We observed similar trends between progression and both pSTAT3 and plasminogen expression modeling the median of all expression values rather than the maximum value (Supplemental Table 1). Stepwise regression analyses yielded results similar to the adjusted models for both the maximum and the median summary expression values, with only pSTAT3 and plasminogen entering the final logistic model (data not shown).

## Discussion

While the biologic mechanisms underlying age-related LI remain unknown, the processes that control postlactational involution have been extensively studied, primarily through investigations in animal models [4]. Postlactational involution proceeds through an initial, reversible stage in which there is widespread apoptotic cell death, followed by an irreversible second stage in which the mammary gland is remodeled to the pre-pregnant state [4]. This process is regulated by complex signaling pathways, including cytokine signaling, apoptosis, inflammatory process, and STAT proteins [14]. Much of the knowledge about the mediators of postlactational involution are derived from animal investigations; while mice do display regression of ductal complexity with age [13], there have been no studies to date to determine whether there are differences in the age-associated regression rate between different mice (or different strains of mice) and, if these differences are present, whether delays in age-associated mammary gland regression in mice may be associated with increased risk of spontaneous mammary cancer (or susceptibility to oncogene- or chemical-induced mammary cancers). In this study, we evaluated expression of a select set of these signaling molecules in TDLUs of clinical BBD biopsies that were judged as either progressive or non-progressive in their LI status to identify any shared pathways that might exist between age-related LI and postlactational involution. We assessed staining using a common digital analysis method that provided consistent application of the objectively defined quantification metrics across the whole sample set. Our analyses collapsed multiple per-TDLU expression values per woman into a single individual-specific summary measure based on the maximum of all values. Two main findings emerged: (1) progression was associated with lower expression of pSTAT3; and (2) progression was associated with higher expression of plasminogen. These findings present the first major advancement in understanding the biological mechanism of age-related LI.

Factors identified as regulators of the two-phase process of postlactational mammary gland involution include transforming growth factor-β3 during the first phase of involution [31] and the IL-6 cytokine family members leukemia inhibitory factor (LIF) in the first phase [32, 33] and oncostatin M (OSM) during the second phase [34]. All of these cytokines activate STAT3, which is essential for the initiation of apoptosis and remodeling following forced weaning [35]; the absence of STAT3 leads to delayed postlactational involution [35, 36]. Conversely, deletion of suppressor of cytokine signaling 3 (SOCS3), which suppresses STAT3 expression, results in premature involution, and increased activation of c-MYC and its pro-apoptotic effectors E2F1, BAX, and p53 [36, 37]. Thus, there is ample evidence for the involvement of the apoptotic cell death machinery downstream of STAT3 activation for postlactational involution. This presents a contrast with our observations for pSTAT3 in age-related LI, as we found that levels of phosphorylated STAT3 are decreased in the tissue of women who will undergo LI relative to the tissue of women with non-progressive LI. One possibility is that the STAT3-activated apoptotic signaling pathway is disrupted in women who do not progress in LI, and levels of phosphorylated STAT3 accumulate as the factors regulating STAT3 continue to signal activation; additional analysis of downstream signaling effectors could provide insight into this possibility. It is also possible that pSTAT3 may regulate other non-apoptotic pathways, which may be present in the aging mammary gland and which may impact LI progression.

The plasminogen cascade of serine proteases is involved in many different processes in many different tissues, primarily due to the ability of this system to regulate pericellular proteolytic activity [38], and dysregulation of this system results in tumor growth and metastasis formation [20]. The involvement of plasminogen in postlactational involution became clear when plasminogen-deficient mice were found to be defective in postlactational involution [21]. In plasminogen knockout mice, the reduction in overall mammary gland size after 5 days of involution was less than one-third the reduction seen in wild-type mice, and the reduction in secretory alveolar volume was almost one-thirtieth the reduction seen in wild-type mice [21]. Although the effect of plasminogen deficiency on postlactational involution is evident, the potential mechanism by which plasminogen loss leads to abnormal postlactational involution is unknown. One possibility that has been advanced is that plasminogen loss results in a failure to sense apoptotic signals [21], which is consistent with the role for plasmin, the activated form of plasminogen, in a variety of apoptotic mechanisms [39], as well as the decreased apoptosis observed in involuting mammary glands in plasminogen knockout mice. It may be that plasminogen is also needed for apoptosis in age-related LI.

An interesting finding in our study is the fact that associations of pSTAT3 and plasminogen with progressive LI strengthen after adjustment for each other in logistic regression models. This is due to the fact that pSTAT3 and plasminogen are positively associated with each other (*r* = 0.37, Fig. 3), and that each is associated with progressive LI (pSTAT3 negatively and plasminogen positively). Because of this complex relationship, failure to account for the collinearity of the two biomarkers in regression models will result in a spurious dilution of association of each with LI progression. This underscores the importance of accounting for inter-relationships between biomarkers when assessing their independent effects on clinical outcomes.

Strengths of the study include the unique tissue resource, which allowed us to define LI progression status in individual women through evaluations of sequential separate biopsies, and to assess correlations of these rates with biomarker expression. Limitations of our findings include evaluation of only a limited number of the many potential pathways implicated in postlactational involution. Immune cells have been implicated as playing critical roles in the postnatal mammary gland development [40]; in particular, pro-inflammatory macrophages have been implicated in the increased breast cancer risk during postlactational involution [41–44], and assessment of immune markers expression and localization vs LI progression status is an important future area of investigation. Additional limitations include potential losses of information and a resulting decrease in statistical power from using a single individual-specific summary measure based on the maximum of all values for a given patient. We had initially intended to fit models that included all TDLU-specific expression values and account for intra-individual collinearity using a generalized estimating equation (GEE) approach within the logistic regression framework. We attempted this using a number of different GEE correlation structures, ranging from a simple exchangeable pattern to an unstructured pattern. Model likelihoods and individual parameter estimates failed to converge in each instance, requiring us to resort to the summary values described herein. Also, the use of the maximum expression biomarker value across all lobules could have introduced a bias, such that women with more lobules would have a greater chance to have a higher maximum value than those with fewer lobules. We mitigated this somewhat by examining no more than 15 lobules per sample. Also, associations using median values, which would not introduce any such bias, were similar in direction to those with maximum values. Finally, all biomarkers are subject to intra-lesion heterogeneity, much like histology. Using the maximum biomarker value across the lesion is similar in rationale to summarizing the benign histology based on the most extreme histologic characteristic, be it non-proliferative disease, proliferative disease without atypia, or atypical hyperplasia. Other limitations include the use of overall staining intensity as our metric, as this may mask critical location-dependent information for some biomarkers; as a consequence, the fact that we did not find significant associations for some of the assessed biomarkers with LI progression does not indicate that no association exists. Finally, none of the *p* values presented in the manuscript account for multiple testing. It is possible, even though the biomarkers are correlated and thus the number of independent tests is smaller than the total number of tests, that accounting for multiple testing would have resulted in non-significant associations. Because of our modest sample size and the fact that this study was somewhat discovery in nature, we chose to present uncorrected *p* values. Certainly, our associations will need to be replicated by others to confirm results.

In summary, we have identified for the first time molecules that may play a role in mediating age-related LI. Further studies of each molecule, both in human tissue and mouse models, will evaluate their validity and begin to address the biological mechanism of age-related LI. Understanding the biological mechanism of age-related LI is likely to provide important insights into why the process of LI is delayed in approximately 40% of postmenopausal women [2], a group with increased risk for breast cancer compared with women who do not have delayed LI.

## Electronic supplementary material

Below is the link to the electronic supplementary material.

Supplementary material 1 (DOCX 15 kb)

Supplementary material 2 (PDF 6653 kb)
