# Supplementary material for: Postlactational involution biomarkers plasminogen and phospho-STAT3 are linked with active age-related lobular involution
Source: Breast Cancer Res Treat. 2017 Jul 27;166(1):133–43. doi: 10.1007/s10549-017-4413-3 (PMC5645446; doi:10.1007/s10549-017-4413-3)
Supplement: Supplementary file 1 — Supplementary material 1 (DOCX 15 kb) [file 10549_2017_4413_MOESM1_ESM.docx]

**Supplemental Table 1.** Associations of progression of involution with biomarker expression, based on median expression across all TDLU measured within an individual.

| Biomarker | OR (95% CI)^1,2^ | p-value^1,2^ | OR (95% CI)^1,3^ | p-value^1,3^ |
| --- | --- | --- | --- | --- |
| CK19 | 0.80 (0.41 – 1.52) | 0.49 | 0.67 (0.30 – 1.40) | 0.29 |
| CK14 | 0.67 (0.32 – 1.32) | 0.25 | 0.78 (0.30 – 1.87) | 0.58 |
| CD44 | 0.88 (0.44 – 1.75) | 0.71 | 1.86 (0.74 – 5.32) | 0.19 |
| MMP9 | 0.90(0.47 – 1.72) | 0.75 | 1.00 (0.48 – 2.17) | 0.99 |
| Tenascin C | 0.63(0.29 – 1.31) | 0.22 | 0.49 (0.17 – 1.32) | 0.16 |
| pSTAT3 | 0.55 (0.26 – 1.11) | 0.10 | 0.29 (0.09 – 0.81) | 0.02 |
| Cmyc | 0.92 (0.49 – 1.71) | 0.80 | 1.36 (0.59 – 3.34) | 0.47 |
| Plasminogen | 1.43 (0.72 – 2.96) | 0.31 | 2.16 (0.89 – 5.87) | 0.09 |
| Ki67 | 0.71 (0.37 – 1.33) | 0.29 | 0.62 (0.26 – 1.42) | 0.26 |
|  |  |  |  |  |

SD, standard deviation; OR, odds ratio; CI, confidence interval.

1. All biomarker values except Ki67 were transformed using a probit (inverse normal) transformation.
2. Adjusted for year of initial biopsy, age at initial biopsy and time between initial and subsequent biopsy.
3. Adjusted for year of initial biopsy, age at initial biopsy, time between initial and subsequent biopsy and all other biomarkers in the table.
