# Supplementary material for: Postlactational involution biomarkers plasminogen and phospho-STAT3 are linked with active age-related lobular involution
Source: Breast Cancer Res Treat. 2017 Jul 27;166(1):133–43. doi: 10.1007/s10549-017-4413-3 (PMC5645446; doi:10.1007/s10549-017-4413-3)

Supplementary Figure 1. Negative controls for each stain. All images are at the same magnification. *Scale bar* 100 μm.

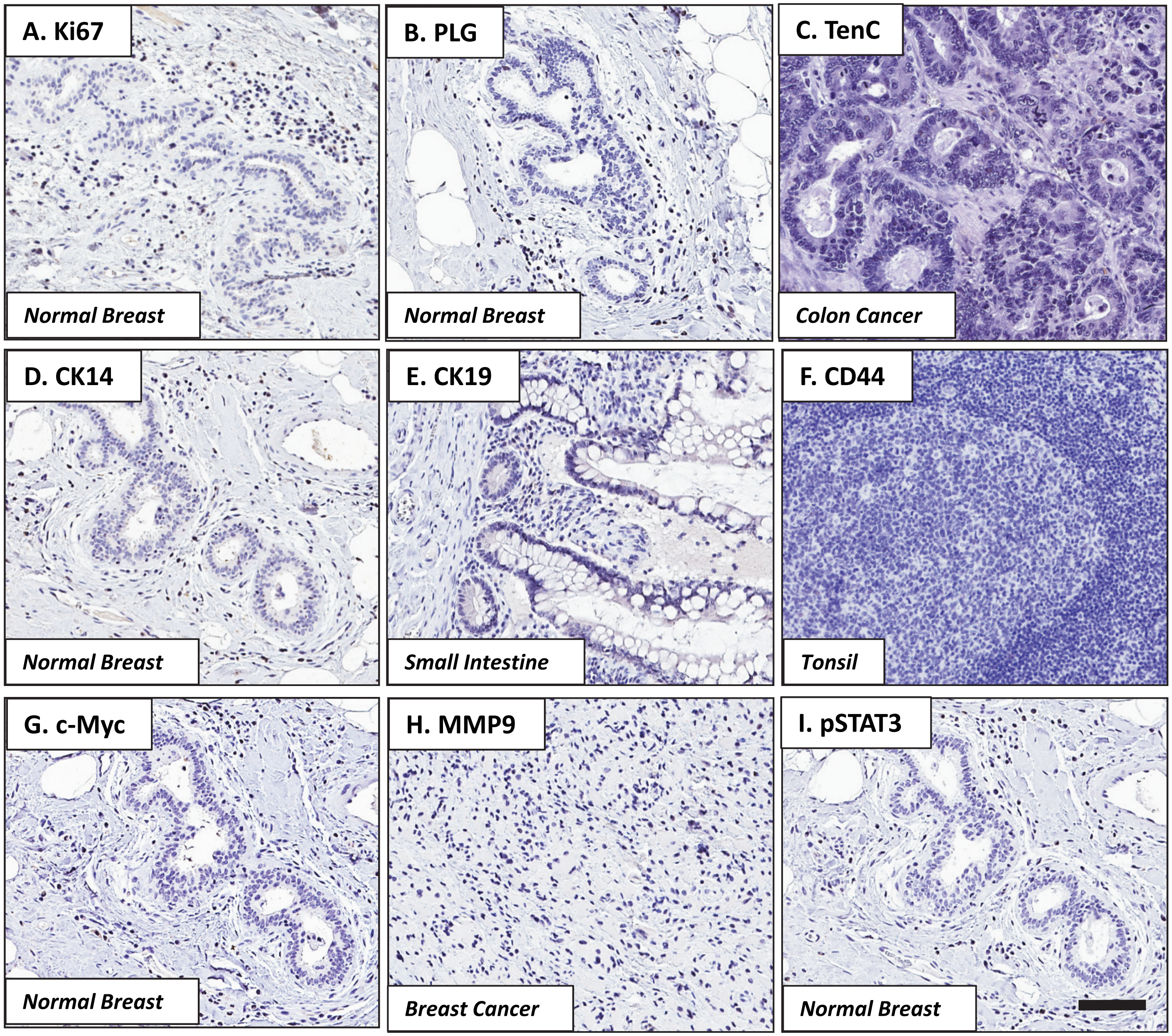

Supplementary Figure 2. Images depicting low/moderate/high expression for Plasminogen (A) and pSTAT3 (B). All images are at the same magnification. *Scale bar* 100 µm.

A. Plasminogen

Low Expression | Moderate Expression | High Expression

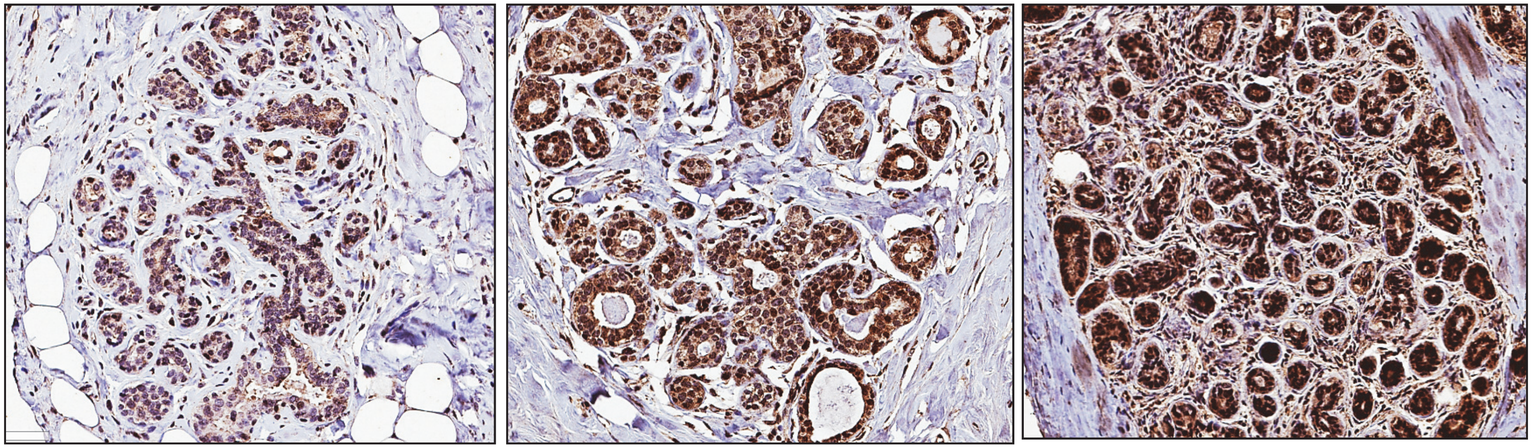

B. pSTAT3

Low Expression | Moderate Expression | High Expression

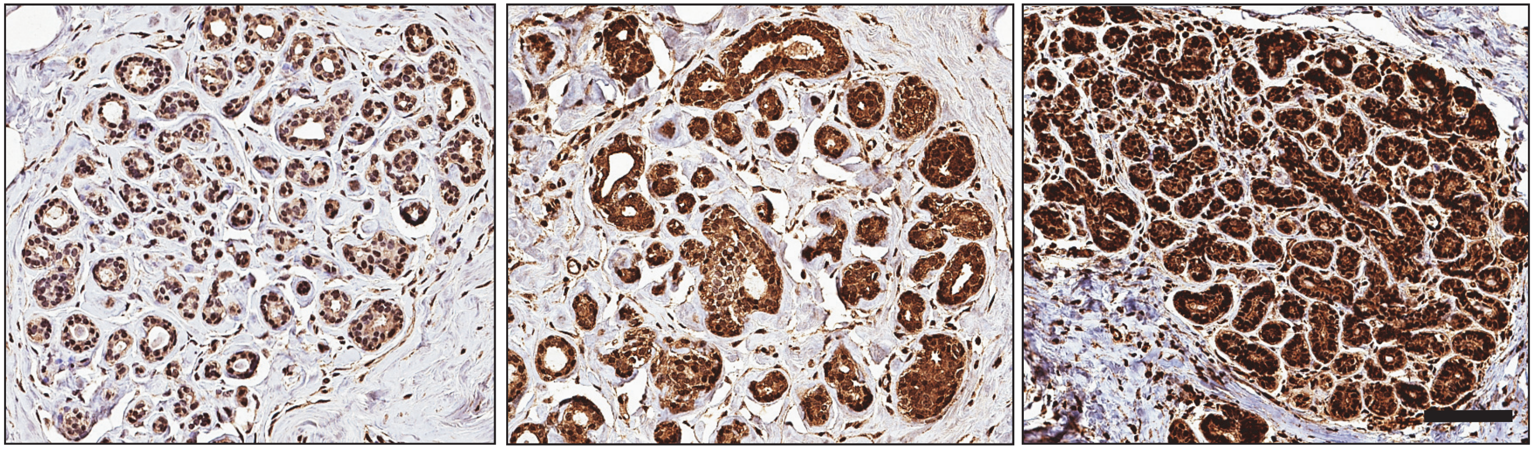

Supplementary Figure 3. Images depicting higher Plasminogen/lower pSTAT3 staining for two patients showing progressive LI (A) and lower Plasminogen/higher pSTAT3 for two cases showing non-progressive LI (B). All images are at the same magnification. *Scale bar* 50 μm.

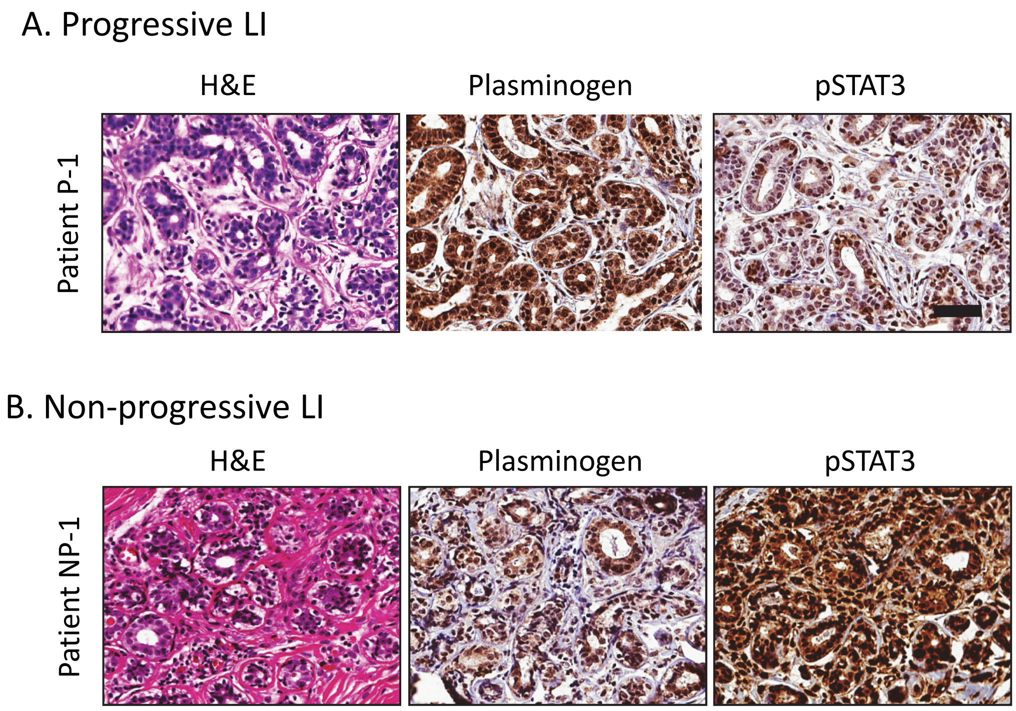

Supplement: Supplementary file 2 — Supplementary material 2 (PDF 6653 kb) [file 10549_2017_4413_MOESM2_ESM.pdf]
